# Supplementary material for: β-Galactosidase-Producing Isolates in Mucoromycota: Screening, Enzyme Production, and Applications for Functional Oligosaccharide Synthesis
Source: J Fungi (Basel). 2021 Mar 19;7(3):229. doi: 10.3390/jof7030229 (PMC8003776; doi:10.3390/jof7030229)
Supplement: Supplementary file 1 [file jof-07-00229-s001.pdf]

Supplementary material

β-Galactosidase-producing isolates in Mucoromycota: screening, enzyme production, and applications for functional oligosaccharide synthesis

Bettina Volford [1], Mónika Varga [1], András Szekeres [1], Alexandra Kotogán [1], Gábor Nagy [1,2], Csaba Vágvölgyi [1], Tamás Papp [1,2], Miklós Takó [1,]*

[1] Department of Microbiology, Faculty of Science and Informatics, University of Szeged, Közép fasor 52, H-6726 Szeged, Hungary

[2] MTA-SZTE "Lendület" Fungal Pathogenicity Mechanisms Research Group, University of Szeged, Közép fasor 52, H-6726 Szeged, Hungary

*Correspondence: tako78@bio.u-szeged.hu; Tel.: +36-62-544-516

List of supplementary data

Table S1. Mucoromycota strains included in the β-galactosidase production screening assays and their activity on X-gal contained medium. The intensity of the blue color is proportional with the enzyme activity.

Figure S1. SDS-PAGE analysis of the crude β-galactosidases. Lane 1 and Lane 2 are *L. ramosa* and *R. pusillus* partially purified β-galactosidases, respectively, obtained after filtration through a Bio-Gel P-6 desalting cartridge (Bio-Rad, Hercules, USA). Lane M: SeeBlue Plus2 SDS-PAGE molecular weight standard (Invitrogen, Carlsbad, USA).

Table S1. Mucoromycota strains included in the β-galactosidase production screening assays and their activity on X-gal contained medium. The intensity of the blue color is proportional with the enzyme activity.

Fungal strains	Code [2]	β-Galactosidase activity [1] *t*(incubation)/day					
		1	2	4	6	8	10
***Lichtheimia* group**							
Lichtheimia brasiliensis	SZMC 23766	NC	NC	NC	NC	NC	NC
Lichtheimia corymbifera	SZMC 11361	++	++++	++++	+++++	+++++	+++++
Lichtheimia corymbifera	SZMC 11367	NC	+	+	++	++	+++
Lichtheimia corymbifera	SZMC 23760	+	+++	++++	++++	++++	+++++
Lichtheimia corymbifera	SZMC 23761	+	+++	++++	+++++	+++++	+++++
Lichtheimia hyalospora	SZMC 11363	+	+++	++++	++++	+++++	+++++
Lichtheimia hyalospora	SZMC 11364	++	++++	++++	+++++	+++++	+++++
Lichtheimia hyalospora	SZMC 23765	NC	++	++	+++	+++	++++
Lichtheimia ornata	SZMC 11370	++	++++	++++	++++	++++	+++++
Lichtheimia ornata	SZMC 11368	+	+++	+++	++++	+++++	+++++
Lichtheimia ornata	SZMC 23764	+	+++	++++	+++++	+++++	+++++
Lichtheimia ramosa	SZMC 11369	++	++	++++	++++	++++	++++
Lichtheimia ramosa	SZMC 11360	+++	+++++	+++++	+++++	+++++	+++++
Lichtheimia ramosa	SZMC 11362	++	++++	++++	+++++	+++++	+++++
Lichtheimia ramosa	SZMC 23762	+	+++	+++	++++	++++	+++++
Lichtheimia ramosa	SZMC 23763	+	++	+++	+++	++++	++++
Lichtheimia sphaerocystis	SZMC 11371	+	+++	+++	++++	+++++	+++++
Lichtheimia sphaerocystis	SZMC 23768	+	+++	++++	++++	++++	+++++

Fungal strains	Code [2]	β-Galactosidase activity [1]					
		t(incubation)/day					
		1	2	4	6	8	10
***Mortierella* group**							
Mortierella alpina	SZMC 11213	NC	NC	NC	NC	NC	NC
Mortierella gamsii	SZMC 11215	NC	NC	NC	+	++	++
Mortierella antarctica	SZMC 11217	NC	NC	NC	NC	NC	NC
Mortierella echinosphaera	SZMC 11251	+	+	+++	++++	++++	+++++
Mortierella exigua	SZMC 11257	NC	NC	+	++	++	++
Mortierella turficola	SZMC 11207	NC	NC	+	++	++	+++
Mortierella gemmifera	SZMC 11201	+	+	++	+++	+++	+++
Mortierella globulifera	SZMC 11209	+	++	+++	+++	++++	+++++
Mortierella indohii	SZMC 11253	NC	+	++	+++	+++	++++
Mortierella capitata	SZMC 11256	NC	NC	NC	NC	NC	NC
Mortierella paraensis	SZMC 11272	NC	NC	NC	NC	NC	NC
Mortierella parvispora	SZMC 11266	NC	+	+	++	+++	+++
Mortierella rishikesha	SZMC 11273	NC	NC	+	++	+++	++++
Mortierella wolfii	SZMC 11243	+	++	+++	+++	+++	++++
Mortierella zychae	SZMC 11212	NC	+	++	++	++	+++
Mortierella epicladia	SZMC 11247	NC	NC	NC	+	+	++
***Mucor* group**							
Mucor amphibiorum	SZMC 12014	NC	NC	NC	NC	NC	NC
Mucor circinelloides	SZMC 20680	NC	NC	NC	NC	NC	NC
Mucor circinelloides	SZMC 12028	+	++	++	+++	+++	++++
Mucor corticolus	SZMC 12031	NC	NC	NC	+	+++	+++

Fungal strains	Code [2]	β-Galactosidase activity [1]					
		t(incubation)/day					
		1	2	4	6	8	10
Mucor corticolus	SZMC 12058	NC	NC	NC	NC	NC	NC
Mucor fragilis	SZMC 0482	NC	NC	NC	NC	NC	NC
Mucor guillermondii	SZMC 12011	NC	NC	NC	NC	NC	NC
Mucor hiemalis f. *hiemalis*	SZMC 12056	+	++	++	++	++	+++
Mucor mucedo	SZMC 0485	NC	NC	NC	NC	NC	NC
Mucor piriformis	SZMC 12077	NC	NC	NC	NC	NC	NC
Mucor plumbeus	SZMC 12070	NC	NC	NC	NC	NC	NC
Mucor plumbeus	SZMC 12635	NC	NC	NC	NC	NC	NC
Mucor plumbeus	SZMC 12023	++	++++	++++	++++	++++	+++++
Mucor racemosus	SZMC 0472	NC	NC	NC	NC	NC	NC
Mucor irregularis	SZMC 23820	NC	NC	NC	NC	NC	NC
Mucor irregularis	SZMC 23821	NC	NC	NC	NC	NC	NC
Mucor racemosus f. *chibinensis*	SZMC 12005	NC	NC	NC	NC	NC	NC
***Rhizomucor* group**							
Rhizomucor miehei	SZMC 11005	++	+++	++++	++++	++++	+++++
Rhizomucor miehei	SZMC 11008	NC	NC	NC	NC	NC	NC
Rhizomucor miehei	SZMC 11014	++	++++	+++++	+++++	+++++	+++++
Rhizomucor miehei	SZMC 11007	NC	+	++	+++	++++	+++++
Rhizomucor miehei	SZMC 11009	NC	NC	NC	+	+	++
Rhizomucor miehei	SZMC 11028	+	+++	++++	++++	++++	+++++
Rhizomucor pusillus	SZMC 11025	+	+++	+++	++++	++++	+++++

Fungal strains	Code [2]	β-Galactosidase activity [1]					
		t(incubation)/day					
		1	2	4	6	8	10
Rhizomucor pusillus	SZMC 11024	NC	NC	+++	++++	++++	++++
Rhizomucor pusillus	SZMC 11022	+	+++	++++	++++	++++	+++++
Rhizomucor pusillus	SZMC 11023	NC	NC	NC	NC	NC	NC
Rhizomucor pusillus	SZMC 11021	NC	+	++	++	+++	+++
***Rhizopus* group**							
Rhizopus arrhizus	SZMC 21290	NC	NC	NC	NC	NC	NC
Rhizopus arrhizus	SZMC 21291	+	+	+	+	+	+
Rhizopus homotallicus	SZMC 13623	NC	NC	NC	NC	NC	NC
Rhizopus microsporus	SZMC 21297	NC	NC	++	+++	++++	++++
Rhizopus microsporus	SZMC 21298	+	++	++	+++	+++	+++
Rhizopus microsporus var. *oligosporus*	SZMC 13622	NC	NC	++	+++	+++	+++
Rhizopus microsporus var. *oligosporus*	SZMC 13619	NC	+	+++	++++	++++	+++++
Rhizopus microsporus var. *rhizopodiformis*	SZMC 13645	++	+++	+++	++++	++++	++++
Rhizopus niveus	SZMC 13625	NC	NC	NC	NC	NC	NC
Rhizopus oryzae	SZMC 0497	NC	NC	NC	NC	NC	NC
Rhizopus oryzae	SZMC 13618	NC	NC	NC	NC	NC	NC
Rhizopus oryzae	SZMC 13635	NC	NC	NC	NC	++	+++
Rhizopus oryzae	SZMC 13643	NC	NC	NC	NC	NC	NC
Rhizopus oryzae	SZMC 0495	NC	NC	NC	NC	NC	NC
Rhizopus oryzae	SZMC 13616	NC	NC	NC	NC	NC	NC

Fungal strains	Code [2]	β-Galactosidase activity [1]					
		t(incubation)/day					
		1	2	4	6	8	10
Rhizopus oryzae	SZMC 13611	NC	NC	NC	NC	NC	NC
Rhizopus oryzae	SZMC 13617	NC	NC	NC	NC	NC	NC
Rhizopus oryzae	SZMC 13634	NC	NC	NC	NC	NC	NC
Rhizopus schipperae	SZMC 21304	NC	NC	NC	NC	NC	NC
Rhizopus stolonifer	SZMC 21295	NC	NC	NC	NC	NC	NC
***Umbelopsis* group**							
Umbelopsis angularis	SZMC 11252	+	++	+++	+++	++++	+++++
Umbelopsis autotrophica	SZMC 11276	+	++	+++	++++	++++	++++
Umbelopsis isabellina	SZMC 11076	+	+	+++	++++	++++	+++++
Umbelopsis isabellina	SZMC 11335	+	+	+++	+++	++++	++++
Umbelopsis isabellina	SZMC 11325	+	++	+++	++++	+++++	+++++
Umbelopsis isabellina	SZMC 11290	+	++	++	++++	++++	+++++
Umbelopsis isabellina	SZMC 11319	+	+	+++	++++	++++	+++++
Umbelopsis isabellina	SZMC 11323	+	++	++	+++	+++	+++
Umbelopsis longicollis	SZMC 11208	+	+++	++++	+++++	+++++	+++++
Umbelopsis ovata	SZMC 22674	+	++	++++	+++++	+++++	+++++
Umbelopsis dimorpha	SZMC 22797	+	+	+	++	+++	+++
Umbelopsis ramanniana	SZMC 11078	+	++	+++	+++++	+++++	+++++
Umbelopsis ramanniana var. *angulispora*	SZMC 11234	+	+++	+++++	+++++	+++++	+++++
Umbelopsis versiformis	SZMC 21866	NC	+	++	++	+++	+++
Umbelopsis versiformis	SZMC 23387	+	+++	++++	+++++	+++++	+++++

Fungal strains	Code [2]	β-Galactosidase activity [1]					
		t(incubation)/day					
		1	2	4	6	8	10
Umbelopsis vinacea	SZMC 11316	++	++	+++	+++	+++	++++
Umbelopsis ovata	SZMC 22674	+	+	+	++	+++	+++

[1] light blue color (+), darker blue color (++), blue color (+++); dark blue color (++++), deep dark blue color (+++++), NC: no blue color. [2] SZMC=Szeged Microbiological Collection.

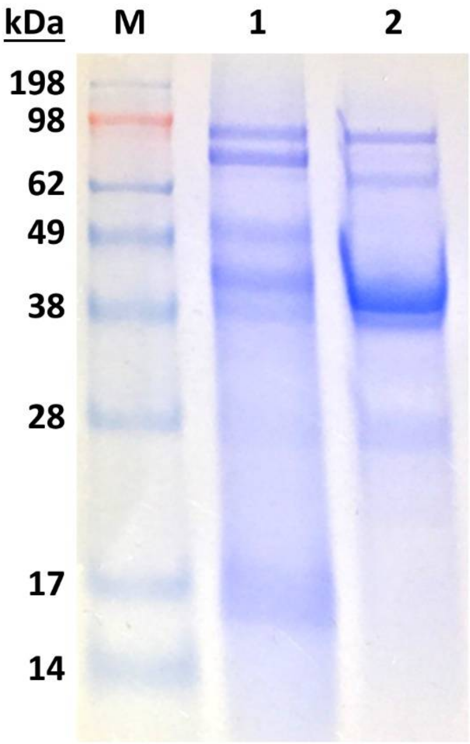

Figure S1. SDS-PAGE analysis of the crude β-galactosidases. Lane 1 and Lane 2 are *L. ramosa* and *R. pusillus* partially purified β-galactosidases, respectively, obtained after filtration through a Bio-Gel P-6 desalting cartridge (Bio-Rad, Hercules, USA). Lane M: SeeBlue Plus2 SDS-PAGE molecular weight standard (Invitrogen, Carlsbad, USA).
